# Exploring the Relationship Between Caffeine Consumption, Caffeine Metabolism, and Sleep Behaviours: A Mendelian Randomisation Study

**DOI:** 10.1111/jsr.70147

**Published:** 2025-07-14

**Authors:** Nilabhra R. Das, Benjamin Woolf, Stephanie Page, Rebecca C. Richmond, Jasmine Khouja

**Affiliations:** ^1^ Population Health Sciences University of Bristol Bristol UK; ^2^ School of Psychological Sciences University of Bristol Bristol UK; ^3^ MRC Integrative Epidemiology Unit University of Bristol Bristol UK; ^4^ MRC Biostatistics Unit University of Cambridge Cambridge UK; ^5^ NIHR Oxford Health Biomedical Research Centre University of Oxford Oxford UK

**Keywords:** caffeine, genetics, Mendelian randomisation, metabolites, sleep

## Abstract

Higher consumption of caffeinated beverages is associated with disturbed sleep patterns. Using genetic variants as proxies for caffeine consumption, caffeine metabolism, and sleep traits, we investigated whether this association reflects a direct effect of caffeine. Genetic variants associated with caffeine consumption (*n* = 407,072), caffeine metabolism (*n* = 9876), chronotype (*n* = 449,734), daytime napping (*n* = 452,633), daytime sleepiness (*n* = 452,071), getting up in morning (*n* = 385,949), insomnia (*n* = 453,379), and sleep duration (*n* = 446,118) identified in individuals from several studies, including the UK Biobank, were used to explore bi‐directional causal relationships between caffeine and sleep using a series of univariable Mendelian Randomisation analyses. We used multivariable Mendelian Randomisation to explore the direct effects of caffeine consumption on sleep behaviours while adjusting for metabolism and vice versa. Higher consumption decreased daytime sleepiness (*β*_univariable_ = −0.044, 95% CI [−0.065, −0.023], *p* < 0.001; *β*_multivariable_ = −0.034, 95% CI [−0.058, −0.009], *p* = 0.010), while faster caffeine metabolism, indicative of less caffeine exposure per beverage consumed, decreased the likelihood of daytime napping (*β*_univariable_ = −0.024, 95% CI [−0.037, −0.011], *p* < 0.001; *β*_multivariable_ = −0.021, 95% CI [−0.042, 0.000], *p* = 0.051). Being an evening person decreased caffeine consumption (*β*_univariable_ = −0.044, 95% CI [−0.078, −0.010], *p* = 0.010). Caffeine consumption/metabolism was not causally related to sleep duration or insomnia. We found no clear evidence for effects of caffeine consumption/metabolism on sleep among non‐current caffeine consumers when assessing possible pleiotropy. Overall, sleep appears to be impacted by caffeine in a way that influences daytime alertness rather than night‐time sleep characteristics. However, the presence of weak instruments for caffeine metabolism and significant heterogeneity warrants further research with larger and diverse samples to better understand the causal pathway between caffeine and sleep.

## 1 Introduction

In the National Diet and Nutrition Survey 2008–2010 (https://www.gov.uk/government/collections/national‐diet‐and‐nutrition‐survey), 95% of individuals in the UK reported caffeine consumption in the form of tea, coffee, energy drinks, chocolate, or other snacks and beverages (Fitt et al. 2013), and in a 2013 study (Mitchell et al. 2014), 85% of US residents reported drinking at least one caffeinated beverage per day. Caffeine may increase alertness and concentration (Treur et al. 2018), although caffeine consumption has also been associated with several adverse effects, including caffeine addiction, poorer sleep quality, and reduced sleep efficiency (Butt and Sultan 2011; Clark and Landolt 2017). A recent review of 58 independent epidemiological studies and randomised controlled trials investigated the association of caffeine consumption with poor sleep including insomnia (Clark and Landolt 2017). The review found that adolescents and adults with high caffeine intake from tea, coffee, and soda were 1.9 times more likely to have trouble sleeping and 1.8 times more likely to feel sleepy in the morning compared to those with lower caffeine intake. Previous studies have highlighted strong associations between poor sleep quality or disturbed sleep and major health issues such as cardiovascular disease and metabolic disorders, diabetes mellitus, as well as respiratory disorders (Zee and Turek 2006). Therefore, a deeper understanding of the impact of caffeine consumption on sleep behaviours could aid public health messaging and facilitate effective dietary advice on caffeine consumption.

The effects of caffeinated product consumption on sleep have previously been attributed to the presence of caffeine in the bloodstream (Cornelis et al. 2016). Following ingestion, caffeine is almost completely metabolised (~95%). Less than 3% of caffeine remains unchanged and is excreted through urine (Kot and Daniel 2008a). Of the 95% of caffeine (chemical name: 1,3,7‐trimethylxanthine [137X]) that is metabolised, 70%–80% is metabolised in the liver by the CYP1A2 enzyme to form paraxanthine (chemical name: 1,7‐dimethylxanthine [17X]) (Begas et al. 2007; Benowitz et al. 1995; Kot and Daniel 2008b). Caffeine exposure (presence of caffeine in bloodstream) for an individual depends on how quickly they metabolise caffeine, which can be quantified using the caffeine metabolite ratio (CMR)—the ratio of paraxanthine to caffeine (Cornelis et al. 2016) (Figure S1). Those with a higher CMR are exposed to less caffeine given a fixed level of intake; therefore, they could be at lower risk of sleep issues due to caffeine exposure (Levy and Zylber‐Katz 1983; Reichert et al. 2021).

The extent to which caffeine affects sleep behaviours in the general population has not been fully established. Traditional epidemiological studies which have been used to explore the influence of caffeine consumption on sleep behaviour may be limited by reverse causality (i.e., where poor sleep and daytime sleepiness in turn influence caffeine intake; Clark and Landolt 2017) as well as residual confounding (Treur et al. 2018) by factors including diet, physical activity, other substances like smoking or alcohol use, and socio‐economic position (Butt and Sultan 2011). Additionally, caffeine consumption measured by self‐reported cups of tea and/or coffee per day may be prone to measurement error, especially as individuals are more likely to misremember their consumption patterns (Abdullah Said et al. 2020). On the other hand, CMR is a more objective measure of caffeine exposure, calculated by profiling blood plasma levels of caffeine metabolites (Cornelis et al. 2016).

**FIGURE 1:**
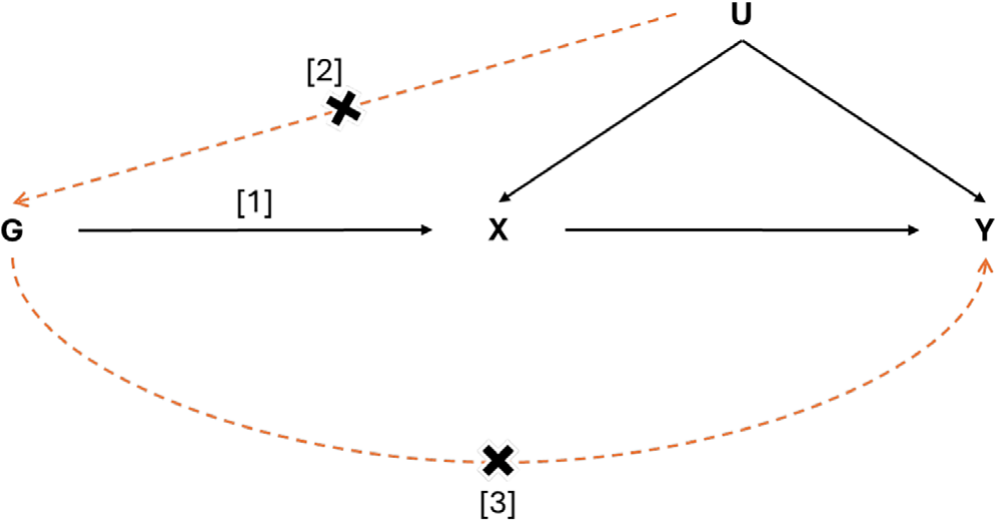
Assumption in Mendelian randomisation (MR) framework. “G” represents the genetic variants, “X” represents the exposure, “Y” represents the outcome, and “U” represents the confounders confounding the relationship between exposure and outcome. “[1]” represents the first assumption (IV1) of the MR framework or “relevance” assumption, “[2]” represents the second assumption (IV2) or “exchangeability” assumption, and “[3]” represents the third assumption (IV3) or “exclusion restriction” assumption.

Mendelian Randomisation (MR) is a causal method that helps mitigate the limitations of observational study design by employing genetic variants as instruments (instrumental variables or IV) for exploring the relationship between exposure and outcome (Smith and Hemani 2014). However, MR results provide valid causal inference only when several MR assumptions are met (Figure 1). The core assumptions are (Skrivankova, Richmond, Woolf, Davies, et al. 2021b):

1. IV1 (“relevance”): instruments must be associated with the exposure,
2. IV2 (“exchangeability” or “independence”): there are no confounders of the association between instruments and the outcome, and
3. IV3 (“exclusion restriction”): instruments are not related to the outcome other than via the exposure.

Genetic variants (single‐nucleotide polymorphisms or SNPs) identified from genome‐wide association studies (GWASs) are used as instruments (i.e., a proxy for actual exposure) in MR. Genetic variants are randomly assigned during meiosis and fixed at conception and cannot be changed as a result of exposure to confounding variables post‐conception. Hence, MR mitigates reverse causation, residual confounding, and tests for causal effects of exposure on outcome without bias given non‐violation of MR assumptions.

A previous study using the MR approach found that higher plasma caffeine affected chronotype (morning/evening preference) but was not linked with other sleep traits, concluding that the associations between caffeine and poor sleep might be driven by shared environmental factors (Treur et al. 2018). However, using caffeine metabolism (substituting consumption) in a univariable MR framework to estimate the effects of caffeine on sleep behaviours, as was done in this previous study, may potentially lead to ambiguous results. Individuals with faster CMR, that is, lower levels of caffeine in blood per beverage, consume more caffeinated products because the effects of caffeine wear off faster.

The investigation of caffeine consumption in an MR framework also remains challenging. A recent study by Woolf et al. (2024) has revealed that 16 SNPs associated with caffeine consumption act through caffeine metabolism, while the other half are linked with behavioural traits like smoking, alcohol consumption, education, and physical activity, which are likely to violate the IV2 assumption. This potentially renders caffeine consumption SNPs as invalid instruments. Moreover, evidence from a previous study suggests that individuals with higher genetically predicted caffeine metabolism rates tend to consume higher amounts of caffeine on average to experience caffeine's alertness‐promoting effects (Zagkos et al. 2024). This may influence the estimated total effects of CMR because the genetic variants associated with faster metabolism may also lead to higher caffeine intake. Hence, estimated total effects of CMR may reflect the biological impact of faster caffeine metabolism combined with the behavioural impact of higher caffeine consumption.

**FIGURE 2:**
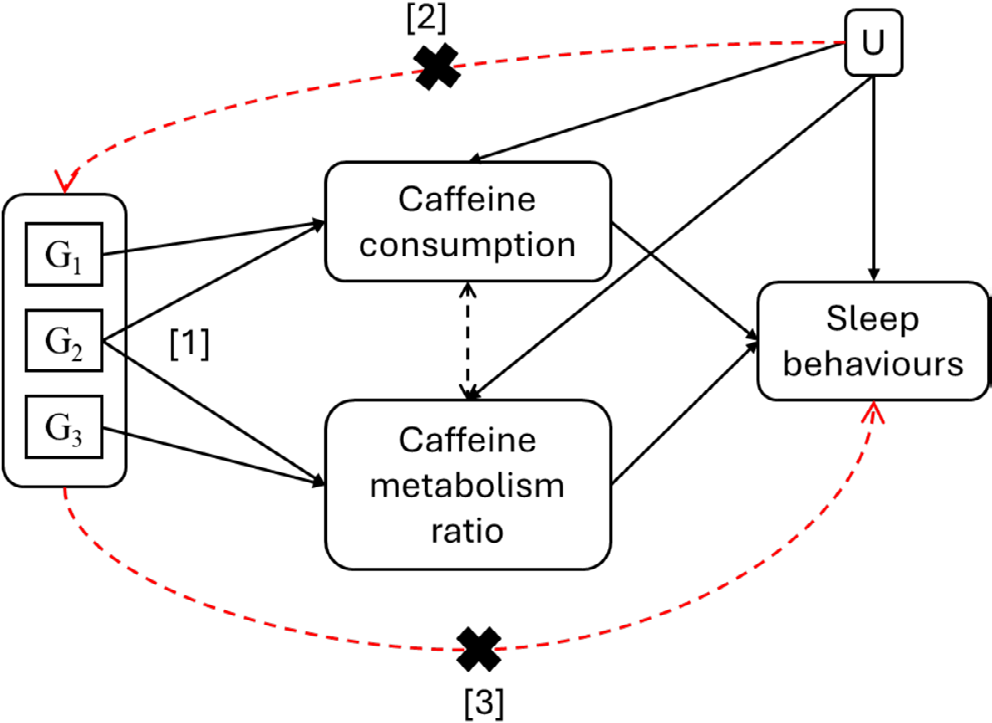
Multivariable Mendelian randomisation (MVMR) framework. “G1,” “G2,” and “G3” represent the genetic variants. “U” represents the confounders confounding the relationship between exposures (caffeine consumption and caffeine metabolism ratio) and outcomes (sleep behaviours). “[1]” represents the first assumption (IV1) of the MVMR framework or “relevance” assumption, “[2]” represents the second assumption (IV2) or “exchangeability” assumption, and “[3]” represents the third assumption (IV3) or “exclusion restriction” assumption.

One alternative is to employ genetic variants for caffeine consumption (cups per day, CPD) and caffeine metabolism (CMR) as instruments in a multivariable‐MR (MVMR) framework. MVMR is an extension of MR that can be used to establish causal effects of two or more related exposures, estimating the direct causal effect of each exposure on an outcome, conditional on the other exposures included in the model (Sanderson et al. 2019). Using this approach, we aimed to explore the direct effects of caffeine consumption on sleep behaviours accounting for caffeine metabolism, and the direct effects of caffeine metabolism on sleep behaviours accounting for consumption (Figure 2).

## 2 Methods

### 2.1 Data Sources

#### 2.1.1 Caffeine Consumption GWASs

Summary statistics were obtained from separate GWASs performed on estimated caffeine consumed from tea, coffee, and combined tea and coffee in a day based on 407,072 individuals of European ancestry in UK Biobank (UKB) (Abdullah Said et al. 2020). Each GWAS was adjusted for age, sex, genotyping array, and the first 30 genetic principal components (PCs) to adjust for population stratification. The UKB and the methods used for each GWAS are detailed in the Supplementary Methods.

#### 2.1.2 Plasma Caffeine and Caffeine Metabolites GWAS

Summary statistics were obtained from a meta‐analysis of six GWASs performed on caffeine metabolism (CMR) and blood plasma caffeine (BPC) based on 9876 individuals of European ancestry (Cornelis et al. 2016). The GWASs were adjusted for smoking status, age, sex, site of the study, fasting status, and family structure and/or study specific PCs of ancestry. The meta‐GWAS is discussed in the Supplementary Methods.

#### 2.1.3 Sleep Behaviour GWASs

Summary statistics on sleep behaviours (chronotype—Jones, Lane, et al. 2019b; daytime napping—Dashti et al. 2021; daytime sleepiness—Wang et al. 2019; getting up in the morning—Jones, Lane, et al. 2019b; sleeplessness/insomnia—Lane et al. 2019; and sleep duration—Dashti et al. 2019) were obtained from GWASs performed on self‐reported traits from approximately 500,000 individuals of European ancestry in UKB available in the Sleep Disorders Knowledge Portal (SDKP) (sleepdisordergenetics.org). Details on the UKB data fields, sample sizes, questions and response choices for sleep behaviours are available in the Supplementary Methods. We used summary statistics from two separate insomnia GWASs (Jansen et al. 2019; Watanabe et al. 2022) which meta‐analysed sleep data from UKB and 23andMe data to cross‐check primary results.

Further, summary statistics from GWAS performed using accelerometer‐derived sleep traits from UKB (Jones, van Hees, et al. 2019a) were obtained for “sleep duration,” “least active 5 hours” (L5) and “most active 10 hours” (M10) (chronotype equivalent), “number of nocturnal sleep episodes” and “sleep efficiency” (aspects of insomnia), and “diurnal inactivity” (consistent with daytime napping) available in the SDKP.

Additionally, we conducted our own GWAS for sleep behaviours stratified by caffeine consumption status (current/non‐current) using UK Biobank Resource under application numbers 16,391 and 81,499. Stratified‐GWASs were performed using the MRC IEU UKB GWAS pipeline (Mitchell et al. 2019) adjusting for sex, genotyping chip, relatedness, and population stratification. Details of the GWAS pipeline are provided in the Supplementary Methods.

### 2.2 Statistical Analysis

#### 2.2.1 Univariable MR

First, we used a two‐sample MR (2SMR) framework with inverse variance weighted (IVW), MR‐Egger, weighted mode, and weighted median models to assess the total causal effects of caffeine consumption (CPD) and caffeine metabolism (CMR) on sleep behaviours. These methods are detailed in Supplementary Methods. Exposures were clumped at a linkage disequilibrium (LD) threshold of *r* ^2^ < 0.001, with a clumping window of > 10,000 kb. Genome‐wide (GW) significant (*p* < 5 × 10^−8^) SNPs were selected and data were harmonised prior to performing MR to ensure SNP‐exposure and SNP‐outcome effects corresponded to the same effect allele (Sanderson et al. 2019; Sanderson 2021; Sanderson et al. 2021). We performed 2SMR using Steiger‐filtering to ensure the correct direction of causality, that is, to confirm that the SNPs used to instrument CMR/CPD are more strongly associated with caffeine metabolism/intake than sleep behaviour (Smith and Hemani 2014). We removed invalid CPD SNPs that were more strongly associated with sleep behaviours than caffeine intake (chronotype: 4; daytime napping: 2; getting up in the morning: 4; insomnia: 3; sleep duration: 2) prior to performing MR analyses. No CMR SNPs were removed from the analyses. Further, we performed the reciprocal MR, using genetic instruments associated with sleep behaviours to evaluate their effects on caffeine consumption and metabolism.

We used an *F* statistic exceeding the conventional threshold of 10 to indicate good instrument strength (IV1) (Burgess and Thompson 2011). A non‐zero intercept in MR‐Egger regression was used to indicate a directional pleiotropic effect (violation of IV2) (Burgess and Thompson 2017). We used funnel plots to assess the presence of horizontal pleiotropy by visually inspecting asymmetry. Cochran's *Q* statistic (Burgess et al. 2017) exceeding the number of SNPs included in each model and the *I* ^2^ statistic (Higgins et al. 2003) were used to indicate heterogeneity. We performed leave‐one‐out analyses to assess where the causal effect was consistent with the removal of individual SNPs. Since GWASs were restricted to individuals of European ancestry while including genetic principal components as covariates, potential confounding due to population stratification (IV2) was minimised.

#### 2.2.2 Multivariable MR

We assessed the role of CMR in the relationship between tea/coffee consumption and sleep behaviours using a two‐sample MVMR (2S‐MVMR) framework. We selected exposure SNPs using a GW significance threshold of *p* < 5 × 10^−8^, then clumped (LD *r* ^2^ < 0.001, clumping window > 10,000 kb) (Sanderson et al. 2019) and harmonised the data (Sanderson et al. 2019; Sanderson 2021) prior to performing analyses using IVW and MVMR‐Egger models. These methods are detailed in the Supplementary Methods.

We used a conditional *F* statistic (cF) over 10 to indicate good instrument strength (Sanderson 2021; Burgess and Thompson 2011). We used weak instrument robust methods—Qhet (Sanderson et al. 2021), debias IVW (Wu et al. 2024), Grapple (Wang et al. 2020), GMM, and IVW ME (Woolf et al. 2022)—to check consistency and robustness in cases of weak instrument strength. Weak instrument robust MR methods are detailed in Supplementary Methods. Cochran's *Q* statistics larger than the number of SNPs included in the model indicate horizontal pleiotropy. We used pleiotropy robust MVMR‐Egger to check the consistency of results (Burgess et al. 2017).

### 2.3 Sensitivity Analyses

Additionally, we performed 2SMR and 2S‐MVMR to assess total effects of CPD and CMR, as well as direct effects of CPD and CMR, on sleep behaviours in non‐current drinkers of tea and coffee. This serves as a negative control to explore violations of IV2 and IV3 assumptions, since we would not expect to observe effects of caffeine consumption and metabolism among individuals who do not drink tea or coffee. We assumed any evidence of causal effects of genetically predicted increased caffeine intake via tea/coffee consumption among non‐current drinkers of tea and coffee would be due to pleiotropy or population stratification, which may bias our primary results.

Furthermore, we performed 2SMR and 2S‐MVMR using accelerometer‐derived sleep traits, multiple GWASs for insomnia, assessing the effects of blood plasma caffeine levels (as well as CMR), and substituting total caffeine consumption with only tea consumption and only coffee consumption to check for consistency in our primary results.

The meta‐analysis GWAS for plasma caffeine and caffeine metabolism did not include UKB data. Hence, there was no overlap in participants between the plasma caffeine and caffeine metabolism GWAS with sleep traits GWASs. However, since the caffeine consumption GWASs were conducted on UKB data, there was overlap in participants with the sleep GWASs. To assess the impact of this, we used MRLap to check for consistency when accounting for sample overlap between exposures and outcomes in two‐sample MR (Mounier and Kutalik 2023).

Analyses were performed in R (version 4.3.3) utilising MendelianRandomization (version 0.10.0), mr.divw (version 0.1.0), MRlap (version 0.0.3.3), MVMR (version 0.4), and TwoSampleMR (version 0.6.4) R packages. Study findings have been reported according to the Strengthening the Reporting of Observational Studies in Epidemiology using Mendelian Randomisation (STROBE‐MR) guidelines (https://www.strobe‐mr.org/) (Table S1) (Skrivankova, Richmond, Woolf, Davies, et al. 2021b; Skrivankova, Richmond, Woolf, Yarmolinsky, et al. 2021a). Lastly, the study protocol was not pre‐registered on any centralised scientific research databases.

## 3 Results

Estimated odds ratios (OR_IVW‐MR_ and OR_IVW‐MVMR_ from IVW MR and IVW MVMR, respectively) for binary traits and estimated betas (*β*_IVW‐MR_ and *β*_IVW‐MVMR_ from IVW MR and IVW MVMR, respectively) for continuous traits are reported in the main text. Presented *β*s correspond to a change of 1 standard deviation (SD) in outcome for 1 SD increase in exposure; ORs also represent the odds per SD increase in the exposure.

### 3.1 Effect of Caffeine on Sleep Behaviours

#### 3.1.1 Instrument Strength

F statistics for CPD (82.04–106.09) and CMR (84.97) and cFs for CPD (10.89–16.58) passed the conventional threshold of 10, suggesting good instrument strength (Tables S2 and S3). However, cFs for CMR (4.71–5.01) indicated weak conditional instrument strength (Table S3). Hence, results from weak instrument robust methods are also presented in the Supporting Information (Figure S8). Cochran's *Q* was greater than the number of SNPs across all sleep traits (Table S4), suggesting the presence of SNP heterogeneity. Similarly, *I* ^2^ indicated that 51%–70% of the variability of MR effect estimates was due to heterogeneity (Table S4). Results for pleiotropy‐robust methods are also presented in the Supporting Information (Figures S2, S4, and S8).

#### 3.1.2 Effects of CPD on Sleep Behaviours

**FIGURE 3:**
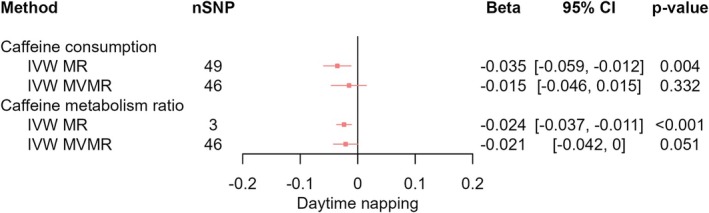
MR results of effects of caffeine consumption and caffeine metabolism ratio on daytime napping. Red squares represent the estimated effect sizes (betas or odds ratios [ORs]) for each individual estimation method. The red horizontal lines represent the 95% confidence intervals (95% CI) for the estimated effects. The black vertical line represents the point of no effect. “nSNP” gives the number of single‐nucleotide polymorphisms (SNPs) used in each estimation method. “IVW MR” gives the total effects estimated using inverse variance weighted (IVW) Mendelian Randomisation (MR). “IVW MVMR” gives the direct effects estimated using multivariable Mendelian randomisation (MVMR).

**FIGURE 4:**
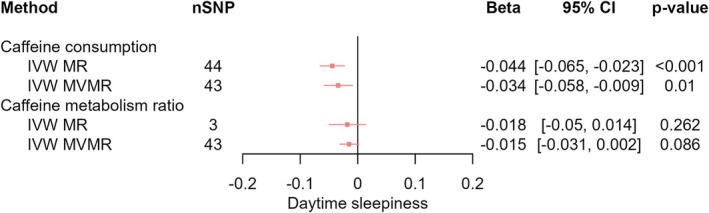
MR results of effects of caffeine consumption and caffeine metabolism ratio on daytime sleepiness. Red squares represent the estimated effect sizes (betas or odds ratios [ORs]) for each individual estimation method. The red horizontal lines represent the 95% confidence intervals (95% CI) for the estimated effects. The black vertical line represents the point of no effect. “nSNP” gives the number of single‐nucleotide polymorphisms (SNPs) used in each estimation method. “IVW MR” gives the total effects estimated using inverse variance weighted (IVW) Mendelian Randomisation (MR). “IVW MVMR” gives the direct effects estimated using multivariable Mendelian randomisation (MVMR).

**FIGURE 5:**
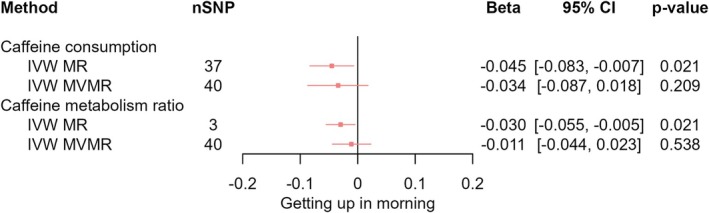
MR results of effects of caffeine consumption and caffeine metabolism ratio on getting up in morning. Red squares represent the estimated effect sizes (betas or odds ratios [ORs]) for each individual estimation method. The red horizontal lines represent the 95% confidence intervals (95% CI) for the estimated effects. The black vertical line represents the point of no effect. “nSNP” gives the number of single‐nucleotide polymorphisms (SNPs) used in each estimation method. “IVW MR” gives the total effects estimated using inverse variance weighted (IVW) Mendelian randomisation (MR). “IVW MVMR” gives the direct effects estimated using multivariable Mendelian randomisation (MVMR).

**FIGURE 6:**
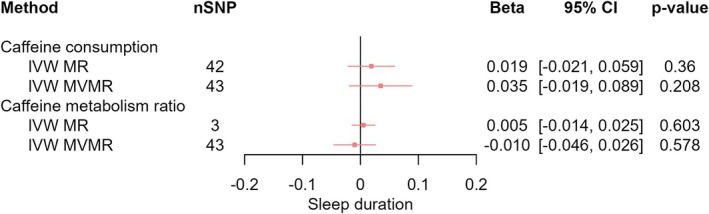
MR results of effects of caffeine consumption and caffeine metabolism ratio on sleep duration. Red squares represent the estimated effect sizes (betas or odds ratios [ORs]) for each individual estimation method. The red horizontal lines represent the 95% confidence intervals (95% CI) for the estimated effects. The black vertical line represents the point of no effect. “nSNP” gives the number of single‐nucleotide polymorphisms (SNPs) used in each estimation method. “IVW MR” gives the total effects estimated using inverse variance weighted (IVW) Mendelian randomisation (MR). “IVW MVMR” gives the direct effects estimated using multivariable Mendelian randomisation (MVMR).

**FIGURE 7:**
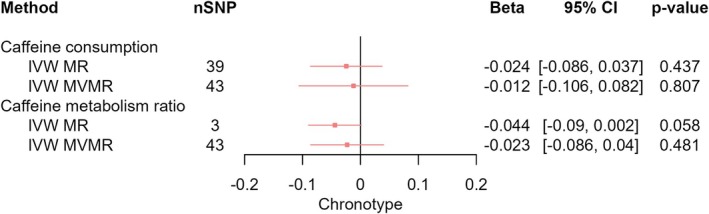
MR results of effects of caffeine consumption and caffeine metabolism ratio on chronotype. Red squares represent the estimated effect sizes (betas or odds ratios [ORs]) for each individual estimation method. The red horizontal lines represent the 95% confidence intervals (95% CI) for the estimated effects. The black vertical line represents the point of no effect. “nSNP” gives the number of single‐nucleotide polymorphisms (SNPs) used in each estimation method. “IVW MR” gives the total effects estimated using inverse variance weighted (IVW) Mendelian randomisation (MR). “IVW MVMR” gives the direct effects estimated using multivariable Mendelian randomisation (MVMR).

**FIGURE 8:**
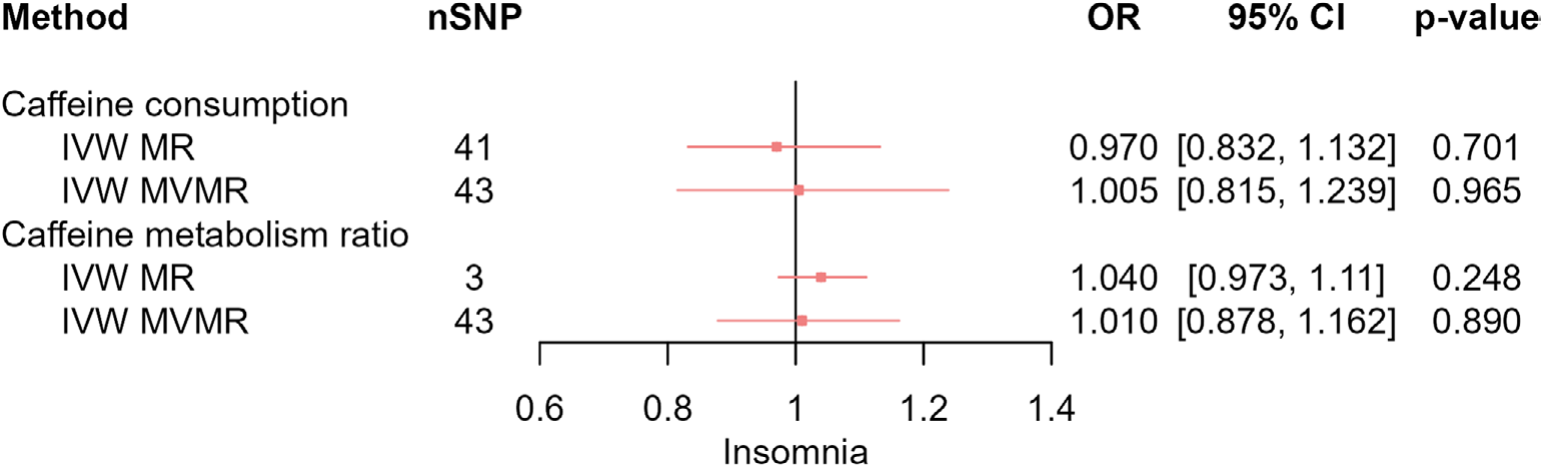
MR results of effects of caffeine consumption and caffeine metabolism ratio on insomnia. Red squares represent the estimated effect sizes (betas or odds ratios [ORs]) for each individual estimation method. The red horizontal lines represent the 95% confidence intervals (95% CI) for the estimated effects. The black vertical line represents the point of no effect. “nSNP” gives the number of single‐nucleotide polymorphisms (SNPs) used in each estimation method. “IVW MR” gives the total effects estimated using inverse variance weighted (IVW) Mendelian randomisation (MR). “IVW MVMR” gives the direct effects estimated using multivariable Mendelian randomisation (MVMR).

In IVW analyses, we found evidence that higher CPD decreased the likelihood of daytime napping (*β*_IVW‐MR_ = −0.035, 95% CI [−0.059, −0.012], *p* = 0.004), which attenuated upon accounting for CMR (*β*_IVW‐MVMR_ = −0.015, 95% CI [−0.046, 0.015], *p* = 0.332) (Figure 3). We found evidence that higher CPD decreased the likelihood of daytime sleepiness (*β*_IVW‐MR_ = −0.044, 95% CI [−0.065, −0.023], *p* < 0.001) which was slightly attenuated upon accounting for CMR (*β*_IVW‐MVMR_ = −0.034, 95% CI [−0.058, −0.009], *p* = 0.010); however, the confidence intervals still excluded the null (Figure 4). We found evidence that higher CPD decreased ease of getting up in the morning (*β*_IVW‐MR_ = −0.045, 95% CI [−0.083, −0.007], *p* = 0.021; *β*_IVW‐MVMR_ = −0.034, 95% CI [−0.087, 0.018], *p* = 0.209) (Figure 5). We found no clear evidence of any total effects of CPD or any direct effects of CPD on sleep duration (*β*_IVW‐MR_ = 0.019, 95% CI [−0.021, 0.059], *p* = 0.360; *β*_IVW‐MVMR_ = 0.035, 95% CI [−0.019, 0.089], *p* = 0.208) (Figure 6), chronotype (*β*_IVW‐MR_ = −0.024, 95% CI [−0.086, 0.037], *p* = 0.437; *β*_IVW‐MVMR_ = −0.012, 95% CI [−0.106, 0.082], *p* = 0.807) (Figure 7), or insomnia (OR_IVW‐MR_ = 0.970, 95% CI [0.832, 1.132], *p* = 0.701; OR_IVW‐MVMR_ = 1.005, 95% CI [0.815, 1.239], *p* = 0.965) (Figure 8). We observed a pattern of attenuated effect estimates across sleep behaviours upon adjusting for CMR, with the exception of the effect of CPD on sleep duration which strengthened when accounting for CMR (*β*_IVW‐MR_ = 0.028, *β*_IVW‐MVMR_ = 0.040), although the confidence intervals included the null (Figure 6). Effect estimates were similar using pleiotropy robust and weak instrument robust MVMR methods across all sleep behaviours presented in the Supplementary Results (Figure S8). Additionally, we found weak evidence that higher CPD decreased ease of getting up in the morning in pleiotropy‐robust univariable MR methods, contrary to our primary results (Figure S2).

#### 3.1.3 Effects of CMR on Sleep Behaviours

In IVW analyses, we found evidence that faster caffeine metabolism (CMR) decreased the likelihood of daytime napping (*β*_IVW‐MR_ = −0.024, 95% CI [−0.037, −0.011], *p* < 0.001; *β*_IVW‐MVMR_ = −0.021, 95% CI [−0.042, 0.000], *p* = 0.051) (Figure 3). We found evidence that faster CMR decreased the ease of getting up (*β*_IVW‐MR_ = −0.030, 95% CI [−0.055, −0.005], *p* = 0.021) which attenuated when accounting for CPD (*β*_IVW‐MVMR_ = −0.011, 95% CI [−0.044, 0.023], *p* = 0.538) (Figure 5). We found weak evidence that faster CMR decreased the likelihood of being an evening person, but again this attenuated when accounting for CPD (*β*_IVW‐MR_ = −0.044, 95% CI [−0.09, 0.002], *p* = 0.058; *β*_IVW‐MVMR_ = −0.023, 95% CI [−0.086, 0.04], *p* = 0.481) (Figure 7). Additionally, we found no clear evidence of any total effects of CMR or any direct effects of CMR on daytime sleepiness (*β*_IVW‐MR_ = −0.018, 95% CI [−0.05, 0.014], *p* = 0.262; *β*_IVW‐MVMR_ = −0.015, 95% CI [−0.031, 0.002], *p* = 0.086) (Figure 4), sleep duration (*β*_IVW‐MR_ = 0.005, 95% CI [−0.014, 0.025], *p* = 0.603; *β*_IVW‐MVMR_ = −0.010, 95% CI [−0.046, 0.026], *p* = 0.578) (Figure 6), or insomnia (OR_IVW‐MR_ = 1.040, 95% CI [0.973, 1.11], *p* = 0.248; OR_IVW‐MVMR_ = 1.010, 95% CI [0.878, 1.162], *p* = 0.890) (Figure 8). We observed patterns of attenuation for the effect of CMR on sleep behaviours after adjusting for CPD.

Total effect estimates as well as direct effect estimates of CMR on other sleep traits were consistent across pleiotropy robust and weak instrument robust methods detailed in the Supplementary Results (Figure S8). However, in our pleiotropy robust univariable MR models, we found evidence that a faster CMR (total effect) increased the likelihood of daytime sleepiness (Figure S4), with some supporting evidence from the MVMR models (Figure S8c).

#### 3.1.4 Effects of Sleep Behaviours on Caffeine Consumption and Metabolism

We next evaluated the effects of sleep behaviours (exposure) on CPD and CMR (outcome). *F* statistics for sleep behaviours were between 40.76 and 52.79 (Table S5). Q statistics were greater than the number of SNPs across all sleep traits, indicating the presence of SNP heterogeneity, which was further supported by high *I* ^2^ statistics (61%–80%) when considering CPD as the outcome (Table S6). However, *I* ^2^ statistics suggested that no amount of the variation (0%) was due to heterogeneity when considering CMR as the outcome (Table S6). Results for pleiotropy‐robust methods are also presented (Figure S4).

**FIGURE 9:**
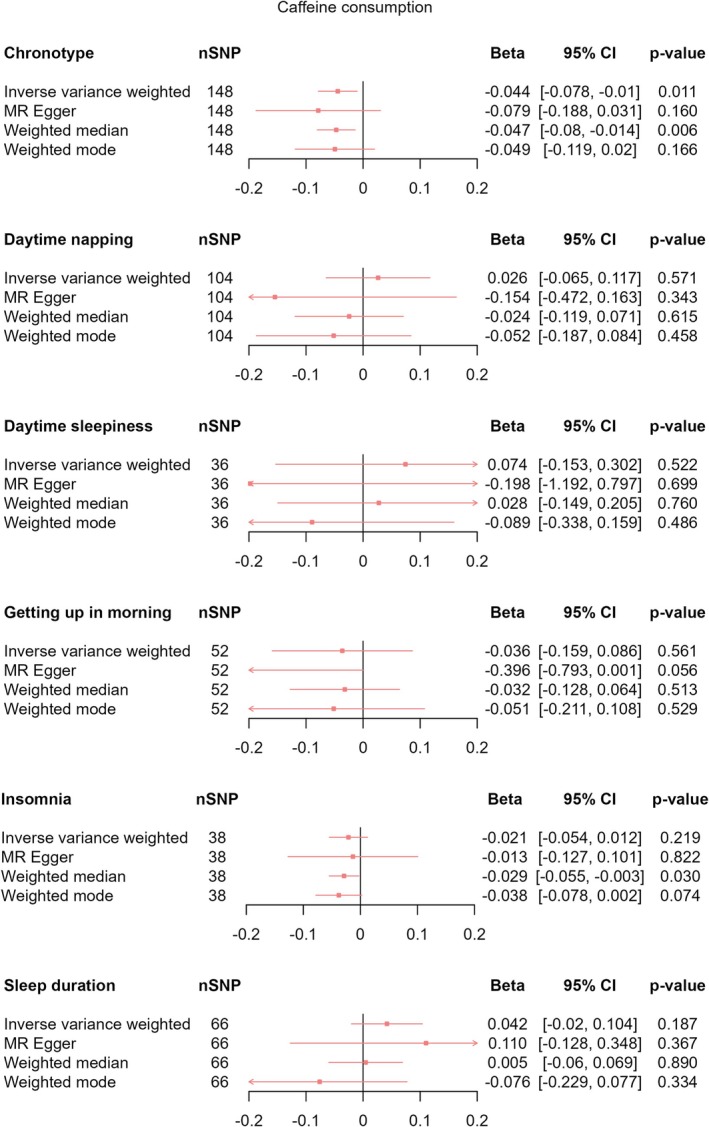
MR results of total effect of sleep behaviours on caffeine consumption. Red squares represent the estimated effect sizes (betas) for each individual estimation method. The red horizontal lines represent the 95% confidence intervals (95% CI) for the estimated effects. The black vertical line represents the point of no effect. “nSNP” gives the number of single‐nucleotide polymorphisms (SNP) used in each estimation method.

**FIGURE 10:**
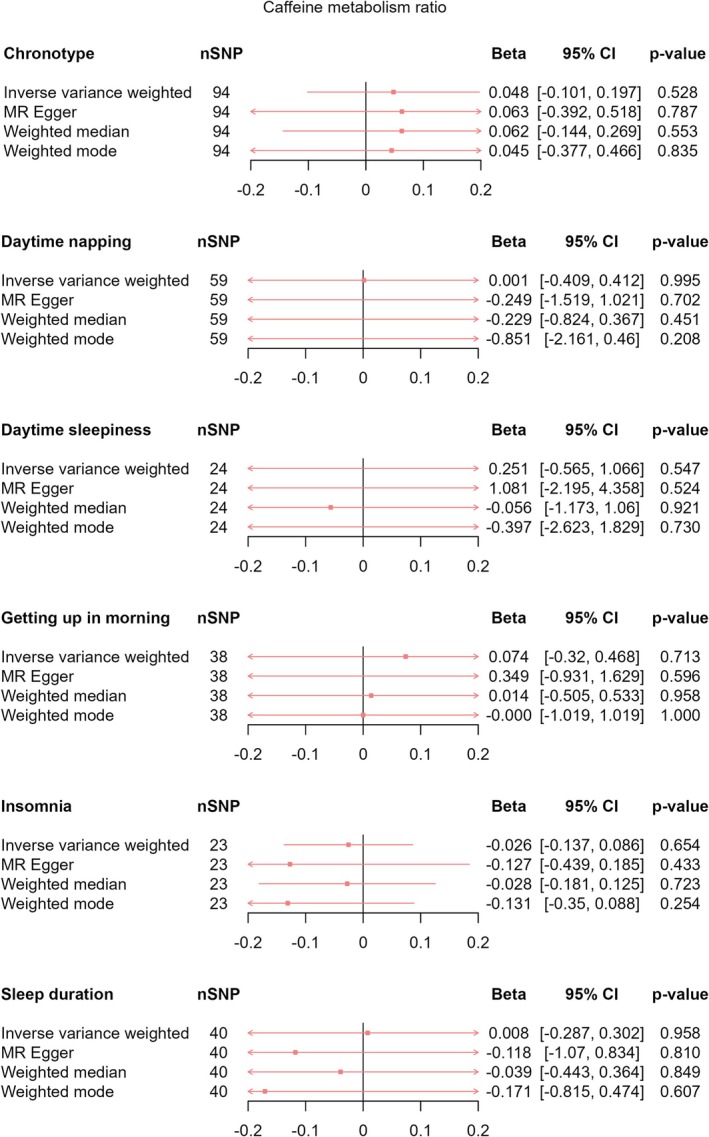
MR results of total effect of sleep behaviours on caffeine metabolism ratio. Red squares represent the estimated effect sizes (betas) for each individual estimation method. The red horizontal lines represent the 95% confidence intervals (95% CI) for the estimated effects. The black vertical line represents the point of no effect. “nSNP” gives the number of single‐nucleotide polymorphisms (SNP) used in each estimation method.

Using IVW‐MR, we found evidence that being an evening person decreased the amount of CPD (*β*_IVW‐MR_ = −0.044, 95% CI [−0.078, −0.010], *p* = 0.011). We observed a consistent direction of effect estimates with wider confidence intervals in pleiotropy robust methods (Figure 9). We found no clear evidence for effects of daytime napping (*β*_IVW‐MR_ = 0.026, 95% CI [−0.065, 0.117], *p* = 0.571), daytime sleepiness (*β*_IVW‐MR_ = −0.074, 95% CI [−0.153, 0.302], *p* = 0.699), ease of getting up in the morning (*β*_IVW‐MR_ = −0.036, 95% CI [−0.159, 0.086], *p* = 0.561), insomnia (*β*_IVW‐MR_ = −0.021, 95% CI [−0.054, 0.012], *p* = 0.219), or sleep duration (*β*_IVW‐MR_ = 0.042, 95% CI [−0.02, 0.104], *p* = 0.187) on CPD (Figure 9), or any effects of sleep behaviours on CMR (chronotype: *β*_IVW‐MR_ = 0.048, 95% CI [−0.101, 0.197], *p* = 0.528; daytime napping: *β*_IVW‐MR_ = 0.001, 95% CI [−0.409, 0.412], *p* = 0.995; daytime sleepiness: *β*_IVW‐MR_ = −0.251, 95% CI [−0.565, 1.066], *p* = 0.547; getting up in morning: *β*_IVW‐MR_ = 0.074, 95% CI [−0.320, 0.468], *p* = 0.713; insomnia: *β*_IVW‐MR_ = −0.026, 95% CI [−0.137, 0.086], *p* = 0.654; sleep duration: *β*_IVW‐MR_ = 0.008, 95% CI [−0.287, 0.302], *p* = 0.958) (Figure 10). Effect estimates were consistent across pleiotropy robust methods (Figures 9 and 10).

Lastly, we did not observe any violations of assumptions in any of our bi‐directional MR frameworks as detailed in Supplementary Results. Sensitivity analysis results and bi‐directional MR assumptions are further detailed in Supplementary Results (Figures S3, S5–S7).

### 3.2 Sensitivity Analyses

For the most part, primary MR and MVMR results were replicated among tea consumers (Figure S15a), coffee consumers (Figure S15b), and current caffeine consumers (Figures S13 and S14), using different insomnia GWASs (Figure S9g,h), and when CMR was substituted for BPC (Figure S10). However, the total effects of CMR (including substitution with BPC) on chronotype were not consistent across our sensitivity analyses, where we found evidence for a positive effect (evening preference) in current caffeine consumers (*β*_IVW‐MR_ = 0.034, 95% CI [0.010, 0.057], *p* = 0.005), and no evidence of any effects when substituted with BPC (*β*_IVW‐MR_ = 0.020, 95% CI [−0.011, 0.051], *p* = 0.209) compared to evidence for a weak inverse effect in our primary analysis (*β*_IVW‐MR_ = −0.044, 95% CI [−0.09, 0.002], *p* = 0.058) (Figure S14a).

For accelerometer‐derived sleep traits, we found evidence of an inverse effect of CPD on diurnal inactivity, which remained following adjustment for CMR (*β*_IVW‐MR_ = −0.051, 95% CI [−0.095, −0.007], *p* = 0.023; *β*_IVW‐MVMR_ = −0.060, 95% CI [−0.111, −0.008], *p* = 0.028) (Figure S9a). Further, we found evidence that higher CPD (unadjusted for CMR) increased sleep efficiency, which slightly attenuated on adjustment for CMR (OR_IVW‐MR_ = 1.007, 95% CI [1.001, 1.013], *p* = 0.019, OR_IVW‐MVMR_ = 1.005, 95% CI [0.999, 1.012], *p* = 0.116) (Figure S9f). However, we did not find any total or direct effects of CPD on L5 time (Figure S9b), M10 time (Figure S9c), number of nocturnal sleep episodes (Figure S9d), and accelerometer‐derived sleep duration (Figure S9e), or any effect of CMR on the accelerometer‐derived sleep traits (Figure S9a–f).

As part of the negative control analysis, we found no clear evidence of any total effects of CPD or CMR, or direct effects of CPD or CMR on the sleep traits among non‐current caffeine consumers (Figures S11 and S12).

Additionally, our primary results were replicated after adjusting for sample overlap between CPD and the sleep traits of interest (Figure S16).

Sensitivity analyses results are further detailed in Supplementary Results (Figures S9, S10, and S13–S16).

## 4 Discussion

In this study, we explored total and direct effects of caffeine consumption measured as cups per day (CPD) on sleep behaviours, adjusting for caffeine metabolism (CMR). Furthermore, we explored the total and direct effects of caffeine measured by CMR on sleep behaviours, adjusting for CPD. In our univariable and multivariable MR analyses, we observed inverse effects of increased caffeine consumption from tea/coffee on daytime napping, daytime sleepiness, and getting up in the morning. We also observed inverse effects of increased caffeine metabolism on chronotype, daytime napping, daytime sleepiness, and getting up in the morning. Additionally, we observed an inverse effect of evening chronotype on caffeine consumption. Consistent with previous MR study findings, we found no clear evidence for effects of caffeine consumption or metabolism on sleep duration or insomnia (Treur et al. 2018; Cheng et al. 2024). Our findings suggest that the effects of caffeine might be more pronounced in daytime alertness phenotypes (chronotype, daytime napping, and daytime sleepiness) over nocturnal sleep phenotypes (ease of getting up in the morning, insomnia, and sleep duration).

Findings that higher caffeine consumption decreased the likelihood of daytime napping, daytime sleepiness, and the ease of getting up are consistent with previous observational studies (Roehrs and Roth 2008; Orbeta et al. 2006). We observed a pattern of attenuation of the effects of caffeine consumption when accounting for caffeine metabolism, suggesting that observed effects of consumed caffeine on sleep behaviours are primarily due to increased circulating caffeine. However, the direct effects of caffeine consumption were often non‐zero, indicating the presence of residual effects from additional constituents of caffeinated drinks such as milk, antioxidants, and sugar, or residual (genetic) confounding by lifestyle/environmental factors as previously suggested (Treur et al. 2018; Woolf et al. 2024). Caffeine tolerance may also interact with the effect of caffeine consumption independently of metabolism (Butt and Sultan 2011), contributing to observed residual effects of CPD. Further, potential horizontal pleiotropy may stem from genetic variations in adenosine (a sleep‐promoting neurotransmitter blocked by caffeine) receptor genes. However, we did not observe any evidence of horizontal pleiotropy in our analyses. We found evidence that a faster caffeine metabolism (CMR) decreased the likelihood of daytime napping, daytime sleepiness, and the ease of getting up (Treur et al. 2018). Individuals with genetic variants associated with faster CMR are more efficient at breaking down and eliminating caffeine (lesser caffeine exposure for a given consumed amount). Faster CMR may lead to more rapid onset and offset of caffeine's stimulant effects, resulting in acute alertness benefits without prolonged exposure to caffeine (less interference with adenosine) that could lead to sleep disturbances—allowing for a smoother transition between periods of wakefulness and sleep, thus reducing the physiological need for daytime napping (O'callaghan et al. 2018).

Similar to Treur and colleagues, we found weak evidence that faster CMR (unadjusted for CPD) decreased the likelihood of being an evening person (Treur et al. 2018). Contrarily, we also found evidence that faster CMR (unadjusted for CPD) increased the likelihood of being an evening person among current consumers. However, these were attenuated to the null in the CPD‐adjusted MVMR models. Moreover, observed effects of CMR in our analyses were in the same (inverse) direction as the effects of CPD.

Contrary to a previous study that employed a similar framework to explore the stimulating effects of nicotine on sleep while adjusting for nicotine metabolism rate (NMR) (Page et al. 2024), our findings indicate contrasting effects of CMR on certain sleep traits (chronotype, daytime napping, daytime sleepiness, ease of getting up in the morning), where the observed CMR effects appear to be in the same direction (negative) as that of CPD, implying that both a faster CMR and a higher CPD increase alertness. Differences in the direction of effects of stimulant metabolism rates could stem from different half‐lives of the stimulants. The stimulating effects of caffeine (half‐life: 5 h) are likely to last longer and potentially into the night compared to nicotine, which has a shorter half‐life (2 h). Moreover, while NMR captures the rate of inactivation of nicotine, CMR is measured as the ratio of paraxanthine to caffeine, where paraxanthine is also an active stimulant. Evidence from functional studies suggests that paraxanthine (the primary metabolite of caffeine) imparts a similar and potentially more efficacious stimulating effect to caffeine (Okuro et al. 2010). The effects of paraxanthine likely contribute to the inverse direction of observed direct effects of CMR in our analyses. A faster CMR leads to a more rapid metabolism of caffeine into paraxanthine, resulting in sustained alertness benefits of paraxanthine without exposure to caffeine for an extended period. Nonetheless, potential physiological effects of paraxanthine are likely to be relatively brief, as its half‐life is about 25% shorter than that of caffeine (Lelo et al. 1986).

Additionally, Treur and colleagues did not observe any effects of sleep behaviours on caffeine consumption (Treur et al. 2018). However, we found evidence that identifying as an evening person decreased CPD.

### 4.1 Strengths and Limitations

This study has several notable strengths. We isolated the effects of caffeine consumption and metabolism on sleep behaviours by using a novel design which includes caffeine metabolism ratio (CMR) as a proxy measure for caffeine in MVMR analyses, while accounting for caffeine consumption (CPD). Additionally, the large sample sizes of GWASs for caffeine consumption and sleep behaviours increase statistical power to detect effects in our analyses, compared with previous MR studies (Treur et al. 2018; Cheng et al. 2024).

However, the study also has some limitations. The small sample size of the caffeine metabolism GWAS relative to caffeine consumption GWAS reduced power to detect direct effects of CMR on sleep behaviours. Additionally, we observed the presence of heterogeneity and pleiotropy in some of our analyses, as well as weak instrument strength for CPD and CMR in our MVMR analyses. However, this was largely overcome with the use of pleiotropy‐, weak instrument‐, and sample overlap‐robust methods (Burgess and Thompson 2011; Burgess and Thompson 2017; Burgess et al. 2017; Mounier and Kutalik 2023), where we observed largely consistent results. Our primary results were also supported by similar effect estimates observed among tea consumers, coffee consumers, and current caffeine consumers. Further, we found no evidence of any effects of CPD or CMR on sleep traits among non‐current caffeine consumers. This analysis serves as a negative control, suggesting evidence against pleiotropy (Sanderson et al. 2019; Sanderson 2021).

Participants misremembering or incorrectly reporting caffeine consumption and sleep behavioural patterns may have introduced measurement error in the self‐reported caffeine consumption and sleep traits (Marques et al. 2024). While we checked the consistency of findings using accelerometer‐derived sleep behaviour data, small sample sizes for accelerometer‐derived sleep trait GWASs resulted in low power to detect effects. However, while MR estimates can be biased by differential measurement error, they are less affected than observational estimates from standard regression (Woolf et al. 2022).

MR and MVMR analyses were performed using GWAS summary statistics based on cohorts primarily of European ancestry. Hence, study findings suffer from a lack of generalisability to populations with non‐European ancestry (Sanderson et al. 2019). Moreover, CPD GWAS included exposure to both caffeinated and de‐caffeinated tea/coffee, which may have diluted GWAS signals. Additionally, there was overlap in participants since GWASs for caffeine consumption and sleep behaviours were conducted using UKB data which may have led to some biased estimates (Hartwig et al. 2016). However, a simulation study has shown that two‐sample MR methods used in a one‐sample setting perform similarly to when used in a two‐sample setting (Minelli et al. 2021). The study further concluded that two‐sample MR methods, except for MR‐Egger, can be safely used for one‐sample MR performed on large biobanks. Further, we used a sample overlap‐robust method (Mounier and Kutalik 2023) to account for the overlap and observed consistent results.

Future studies should utilise larger GWAS on CMR to cross‐check our study findings. UKB is a non‐representative study with about 5.5% participant response rate (Stamatakis et al. 2021). Future assessment of the effects of caffeine on sleep traits should be conducted on studies other than UKB to validate our findings. GWASs based on individuals of non‐European ancestry should be used in future studies to improve generalisability of our study findings or potentially reveal evidence for differences in the effect of caffeine on sleep behaviours between ancestries. Finally, using GWASs performed on caffeinated coffee consumption might further strengthen evidence for effects. Although we observed consistent results in our sensitivity analyses separating out tea and coffee consumption (contrary to our primary analyses using combined tea and coffee consumption), the inclusion of decaffeinated coffee in the coffee consumption measurement may have resulted in a slight underestimation of the observed effects of caffeine.

### 4.2 Implications

Exploring the relationship between sleep and caffeine consumption is important for informing public health guidelines for caffeine consumption and sleep hygiene, while knowledge of genetic predisposition to caffeine metabolism could improve personalised advice regarding how caffeine consumption might affect sleep. Our findings support the role of caffeine on certain sleep behaviours (specifically daytime napping and daytime sleepiness), which could have important implications for public health initiatives aimed at better sleep hygiene, diet, and overall quality of life. However, the limited evidence for a (long‐term) effect of caffeine consumption and metabolism on sleep duration and insomnia indicates that the stimulating effects of caffeine are typically acute, and suggests that the association between habitual caffeine consumption and poor sleep seen observationally may be attributed to residual confounding by shared environmental factors.

## 5 Conclusion

In conclusion, we found evidence for effects of higher caffeine consumption levels on certain sleep behaviours (decreased daytime napping and daytime sleepiness, and to a lesser extent, decreased ease of getting up), and evidence of faster caffeine metabolism on chronotype (morning preference), and decreased daytime napping and ease of getting up. In our MVMR analyses, we found evidence for effects of higher consumption levels on reduced daytime sleepiness and evidence for effects of faster metabolism on reduced daytime napping and daytime sleepiness, suggesting that caffeine plays an important role in determining daytime alertness. A higher caffeine metabolism rate may allow an individual to experience caffeine's alertness‐promoting effects within a shorter period of time. Thus, sleep homeostasis is not disrupted for an extended period of time. This, in turn, could reduce the need for compensatory sleep which is experienced by individuals with slower caffeine metabolism. Our findings suggest that faster metabolism of caffeine may not result in reduced stimulating effects when accounting for the amount of caffeine consumed per day. Since the stimulating effects of caffeine may be sustained by paraxanthine, which is the primary metabolite of caffeine, further exploration of the impact of the metabolites of caffeine on sleep traits is warranted. Moreover, we observed some residual effects of caffeine consumption after adjusting for caffeine metabolism, suggesting effects due to additional constituents of tea/coffee, or lifestyle/environmental factors on sleep. We found no clear evidence for effects of caffeine consumption or metabolism ratio on sleep duration or insomnia. Finally, we observed effects of being an evening/morning person on caffeine consumption levels, suggesting the presence of a sleep–caffeine–sleep behavioural cycle. Our findings suggest that the observed relationships between genetically predicted caffeine consumption, caffeine metabolism, and daytime alertness traits can potentially aid in catering dietary advice to individuals suffering from sleep disorders. However, the weak strength of the caffeine metabolism instruments could have affected the reliability of our results and biased our results towards the null. Although we employed multiple weak instrument‐robust methods to improve the robustness of our findings, we suggest that future studies use better‐powered GWASs to validate them.

## Author Contributions

**Nilabhra R. Das:** investigation, writing – original draft, visualization, writing – review and editing, software, formal analysis. **Benjamin Woolf:** investigation, methodology, validation, writing – review and editing, software, resources, supervision. **Stephanie Page:** investigation, writing – review and editing, validation, software, resources. **Rebecca C. Richmond:** conceptualization, investigation, funding acquisition, validation, writing – review and editing, project administration, resources, supervision. **Jasmine Khouja:** conceptualization, investigation, methodology, validation, writing – review and editing, project administration, resources, supervision.

## Conflicts of Interest

The authors declare no conflicts of interest.

## Supporting information


**Data S1.** Supporting Information.

## Data Availability

The data that support the findings of this study are openly available in GWAS Catalog at https://www.ebi.ac.uk/gwas/.
